# Systemic Heparinisation in Laparoscopic Live Donor Nephrectomy

**DOI:** 10.1155/2013/138926

**Published:** 2013-12-16

**Authors:** Charlotte Crotty, Yasmin Tabbakh, Sarah A. Hosgood, Michael L. Nicholson

**Affiliations:** Transplant Group, Department of Infection, Immunity and Inflammation, Leicester General Hospital, University of Leicester, Leicester LE5 4PW, UK

## Abstract

*Introduction*. Systemic heparinisation is advocated during laparoscopic live donor nephrectomy (LDN) as a preventative measure against renal vascular thrombosis during the warm ischaemic interval. This study compares the outcome with and without the administration of systemic heparinisation. *Methods*. A retrospective analysis was performed on 186 consecutive LDN patients between April 2008 and November 2012. Systemic heparin (2000–3000 IU) was administered intravenously to donors (hep *n* = 109). From January 2010, heparin was not used systemically in this group of LDN (no hep *n* = 77). Outcome measures included donor and recipient complications, initial graft function, and 12 month graft survival. *Results*. The demographics of both heparinised and non-heparinised donors were similar. The warm ischaemic time (WIT) was comparable in both groups (WIT; hep 5 ± 3 versus no hep 5 ± 3 minutes; *P* = 1.000). There was no difference in complication rates, no episodes of graft thrombosis, and no incidences of primary nonfunction in either group. Delayed graft function occurred in 4/109 and 1/77 (3.6% versus 1.2%; *P* = 0.405) and there was no significant difference in graft survival (*P* = 0.650). *Conclusion*. Omitting systemic heparinisation during laparoscopic donor nephrectomy is a feasible and safe approach that does not compromise donor or recipient outcome.

## 1. Introduction

Minimally invasive techniques of surgery for live donor nephrectomy have been rapidly adopted across the UK. Unquestionably this has helped to increase the number of live donor kidney transplants [1]. Kidneys donated by living donors accounted for approximately 36% of all transplants performed in the UK in 2011-2012 [1]. The pure laparoscopic approach uses small incision sites which results in less postoperative pain, reduced hospital stay, improved cosmetics, and earlier return to work than the traditional open technique [2, 3]. This has reduced many of the disincentives associated live kidney donation.

During laparoscopic live donor nephrectomy (LDN) the kidney endures a period of warm ischaemic injury before it is retrieved and flushed with cold preservation solution [4]. Systemic heparin has been advocated during laparoscopic live donor nephrectomy as a preventative measure against intra-renal microthrombi formation during the warm ischaemic interval [5]. However, this subjects the patients to an increased risk of haemorrhage. Protamine sulphate can be used to reverse the effects of heparin but is associated with anaphylactic reactions and pulmonary hypertension [6, 7]. Systemic heparin was previously used for LDN in Leicester but in 2010 the protocol was changed and heparin was not administered. The aim of this study was to examine donor and recipient outcomes associated with or without the administration of systemic heparin during LDN.

## 2. Patients and Methods

### 2.1. Patients

A retrospective analysis was performed on 219 consecutive patients undergoing LLDN from April 2008 to November 2012. Three donors were converted from laparoscopic surgery to an open procedure due to a complication during surgery; however all 3 conversions were carried out before heparin was administered and these cases were consequently excluded from the study. Thirty patients were also excluded due to lack of completed documentation. Therefore, 186 LDN were analysed in this study. All LDN were performed by the same consult transplant surgeon (MLN).

Patient's notes and computerised records were manually assessed for donor and recipient complications, including complications throughout the operative procedure and graft function of the recipient. Graft outcome measures were collected up until 12 months after transplant. All donors who underwent LDN between April 2008 and December 2010 received systemic heparin (*n* = 109). From December 2010 the remaining donors in the series did not receive intraoperative systemic heparin (*n* = 77).

### 2.2. Donor Management

All donors received the same postoperative care. In brief this involved 15-minute blood pressure monitoring for the first 2 hours post operatively, followed by 30-minute observations for the next hour and then hourly for the next 4 hours. Subsequently observations were then taken 4 hourly until discharge. Haemoglobin levels were measured preoperatively and then daily until discharge.

### 2.3. Surgical Techniques and Systemic Heparinisation Protocol

The surgical team made a decision about which kidney to remove based on the result of the split function renal test and the vascular anatomy of the kidney, as demonstrated by spiral Ct angiography computed tomography (CT scan). The laparoscopic surgical procedure was consistent throughout this cohort of 186 patients. A pure laparoscopic, nonhand assisted procedure was used throughout.

A 4-port transperitoneal access was used. Kidneys were extracted via a pfannenstiel incision (6–8 cm), using a fully transperitoneal approach. Two 10 mm ports were used; one placed close to the umbilicus and the other in the ipsilateral iliac fossa. Five mm ports were placed in the epigastrium and the lumbar region. The renal artery was secured with a linear cutting stapler or lockable silastic clips (Weck, Hem-o-lok Closure System, Teleflex medical, NC, USA). The renal vein was divided after controlling with Hem-o-lok clips. Systemic heparin (2000–3000 IU) was administered intravenously to donors 5 minutes prior to arterial clamping.

### 2.4. Outcome Measures

Donor and recipient demographics and the incidences of intra- and postoperative complications in the donor and recipient were assessed. In the recipient, the incidences of graft thrombosis, graft function, and graft survival were recorded. The total ischaemic time was defined from the start of arterial clamping of the donor vessels to reperfusion of the kidney.

Recipient graft function was measured daily using levels of serum creatinine, and eGFR on day 7, 1 month, and 12 months after transplant.

Delayed graft function (DGF) was defined as any form of renal replacement therapy (RRT) needed in the first 7 days after transplant. Acute rejection was diagnosed by histopathological examination of a renal biopsy and treated with 3 × 0.5 grams methylprednisolone for 3 consecutive days. Resistant rejection was treated with antithymocyte globulin (ATG). Graft and patient survival were monitored up to 12 months after transplant.

### 2.5. Statistics

Statistical analysis was performed using an integrated measurement using Excel (Microsoft, Reading) and Graph Pad Prism 5 (Graph Pad Instat, San Diego, CA). Results were displayed as mean ± standard deviation. Mean data was compared using the appropriate *t*-test or contingency test (Fisher's exact). *P* ≤ 0.05 was considered to be statistically significant.

## 3. Results

### 3.1. Demographics 

Donor and recipient demographics are outlined in Table 1. There was no significant difference in the donor demographics between the groups. There was a similar amount of right and left kidneys donated in each group (*P* = 0.386). More kidneys in the heparin group had multiple arteries compared to the nonheparinised group (*P* = 0.027). Several kidneys in each of the groups had dual renal veins (*P* = 0.473).

### 3.2. Intraoperative and Postoperative Outcomes

#### 3.2.1. Donor

There was no significant difference in the duration of warm ischaemia (heparin 5 ± 3 versus nonheparinised 5 ± 3 min; *P* = 1.000) (range 1 to 13 min versus 2–8 min) or in the total ischaemic time (heparin 306 ± 80  versus nonheparinised 295 ± 60 min; *P* = 0.189) between the groups. The warm ischaemic time was significantly longer in kidneys with multiple arteries compared to those with single vessels (6 ± 2.7 versus 4.0 ± 1.3 min; *P* = 0.0001).

There were no intra- or immediate postoperative complications in either of the groups associated with bleeding. There was no significant difference in haemoglobin levels between the groups pre- or postoperative (*P* > 0.05; Table 2). Levels fell significantly day 1 postoperatively in both groups and remained stable until discharge (Table 2).

Patients in the heparin group stayed in hospital significantly longer compared to those in the nonheparinised group (heparin 5 ± 1  versus nonheparinised 4 ± 1 days; *P* = 0.001).

#### 3.2.2. Recipient

The anastomosis time was 26 ± 6 minutes in the heparin group and 30 ± 8 minutes in the nonheparinised group (*P* = 0.0001). There was no significant difference in the anastomosis time in kidneys with single or multiple arteries (28 ± 7.0 versus 27 ± 8.0 min; *P* = 0.091).

There were no episodes of graft thrombosis or primary nonfunction in either group. In the heparinised group six recipients received blood transfusions, compared to three in the nonheparin group (*P* = 0.740).

Three recipients in the heparin group returned to theatre for reexploration. One was due to bleeding from the renal bed and the other two for ureteric complications. Of the three recipients in the nonheparinised group who returned to theatre for reexploration, one was for a clot in the superficial layer, one for washout of haematoma and exploration of transplant wound. The final was due to a ureteric obstruction.

**Figure 1:**
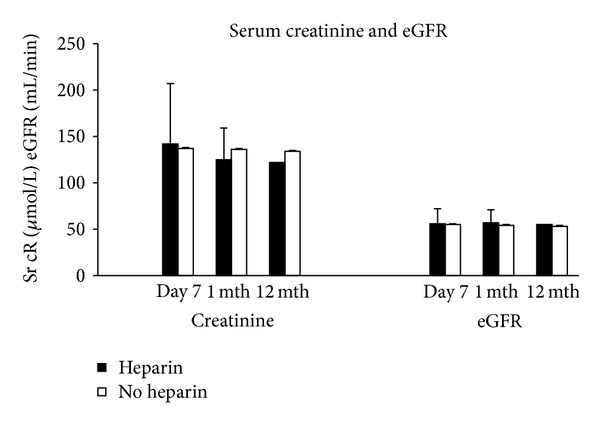
Recipient serum creatinine and eGFR levels in the heparin and nonheparinised groups day 7, 1 month after and 12 months after transplant.

Delayed graft function occurred in 4/109 (3.6%) of heparinised patients and 1/77 (1.2%) of the nonheparinised group (*P* = 0.405). Day 7, 1 month, and 12 months serum creatinine and eGFR levels were not significantly different between the groups (*P* > 0.05; Figure 1).

There was a similar incidence of acute rejection in the two groups (hep 15% versus nonhep 21%; *P* = 0.326).

Graft survival at 12 months was similar, 97.2% (hep) versus 98.7% in the nonheparinised group; (*P* = 0.650). Patient survival at 12 months was 98.2% (hep) versus 96.1% (nonhep; *P* = 0.650). Three grafts failed in the heparin group within the first 12 months. One due to recurrence of primary end-stage renal disease, and two due to rejection that did not respond to treatment. The single graft loss in the nonheparinised group was due to rejection within the first 3 months after transplant. There were 2 patient deaths in the heparin group. One due to a cardiac arrest 3 days after transplant and the other due to ischaemic bowel leading to sepsis 7 days after transplant. In the nonheparinised group one patient died due to adenocarcinoma of the lung, another due to a cerebral head trauma secondary to an epileptic episode, and the third due to a hypoxic brain injury at 11 months after transplant.

## 4. Discussion

Patient safety is paramount in any surgical procedure. However, LDN is a unique situation because it exposes an otherwise healthy patient to the risks of surgery entirely for the benefit of another person. LDN has been shown to be a less minimally invasive alternative compared to open donor nephrectomy [2, 3]. It has had a significant impact on living donor renal transplantation and the number of operations being performed has dramatically increased [1].

The major concern associated with LDN has been the prolonged warm ischaemic time due to the time taken to remove and flush the kidney once the renal vessels have been clamped and secured. This increases the risk of intrarenal microthrombi formation, which could impair graft function leading to delayed graft function (DGF) or cause graft loss [5]. In renal transplantation rates of arterial thrombosis are reported to range between 0.2 and 7.5% and venous thrombosis 0.1 and 8.2% [8]. Systemic heparinisation is routinely used to avoid these complications. However, there are no guidelines on the use of heparin during LDN in the UK.

The duration of warm ischaemia varies significantly between centres in live kidney donation (range 2 to 17 minutes), although this is reported to have no adverse effect on short term graft outcome [4]. The average warm ischaemic time in this present study was 5 minutes and ranged from 1 to 13 minutes. Kidneys with multiple vessels had a longer warm ischaemic time. Simforoosh et al. [9] reported a prolonged warm ischaemic time up to 10 minutes during LDN with no adverse effect on graft function or survival. However, all donors received an intravenous dose of 5,000 IU of heparin 30 minutes before arterial clamping. There are several studies where the warm ischaemic times fell under 5 minutes that reported no adverse effects in not using systemic heparin. In a retrospective analysis of 119 patients, in which the warm ischaemic times were just under 3 minutes Friedersdorff et al. found that 3 heparinised donors suffered moderate postoperative haemorrhage [10]. However, although their complication rates were low they commented that perhaps these numbers could have been reduced by omitting systemic donor heparinisation. There were no reported complications due to bleeding in the donors in this present study; therefore this adds to the evidence that systemic heparinisation is not an added advantage during LDN.

In addition to the increased warm ischaemic interval other factors during LDN can also contribute to complications. These may also advocate the use of heparinisation. The left kidney is the preferred choice for LDN due to the longer renal vein. The shorter renal vein in the right kidney has been associated with increased incidence of renal vein thrombosis [11]. However, as the laparoscopic approach is becoming more widely used, recent evidence suggests that the risk is low. Multiple vessels also can increase the risk of complications. In the past, multiple vessels were discouraged due to technical difficulty, prolonged ischaemic time, the risk of segmental infractions, and ureteric complications [12–14]. Nonetheless, with experience, the safety and feasibility of using kidneys with multiple arteries is being increasingly reported [11, 15]. Furthermore, the anastomosis time was not significantly increased in kidneys with multiple vessels.

In this present series, the patients were well matched with a comparable ratio of right and left kidneys donated. There was a higher incidence of multiple arteries in the patients receiving heparin. Nonetheless, the complication rate was extremely low with no incidences of thrombosis, only 3 recipients in each group requiring reexploration and no significant difference in graft function or survival. Delayed graft function (DGF) occurred in 3.6% versus 1.2% in the nonheparinised group. These rates of DGF fall within ranges reported in literature sources from other worldwide live donor transplant centres [16, 17]. Furthermore, graft function and survival were similar.

Protamine Sulfate can be used to reverse the anticoagulation effects of heparin before completion of the operation. Nonetheless, it has been linked with severe complications and is not commonly used during LDN and was not used in this study [7]. There was an expected fall in donor haemoglobin levels the day after the nephrectomy in both groups of patients. Thereafter, levels remained stable. None of the donors in this series required a blood transfusion.

Three donors were converted to open surgery due to complications during surgery. They were excluded from the analysis because the complications arose before heparin was administered. Conversion to open surgery was a necessary step to prevent any further complications. Hospital stay was increased by one day in donors receiving heparin compared to those without. Systemic heparinisation was used early in the series and since this time there has been a change in practice and a more rapid recovery protocol in place to enhance a faster recovery and reduce hospital stay.

In conclusion omitting systemic donor heparin during LDN is a feasible and safe approach that does not adversely compromise donor or recipient outcomes.

## Figures and Tables

**Table 1:** Donor and recipient demographics, left and right kidney, and renal vasculature.

	Heparin	No heparin	*P* value
Donor			
Age (yr)	47 ± 12	45 ± 12	1.000
Gender M : F	44 : 65	28 : 49	0.648
Left kidney	92 (84.4%)	68 (88.3%)	0.386
Right kidney	17 (15.6%)	8 (10.4%)	0.386
Single artery	80 (73.4%)	68 (88.3%)	
Two arteries	25 (22.9%)	9 (11.7%)	0.027
Three arteries	4 (3.7%)	0	
Single vein	103 (94.5%)	75 (97.4%)	0.473
Dual veins	6 (5.5%)	2 (2.6%)	
Recipient			
Age (yr)	47 ± 12	43 ± 14	0.659
Gender M : F	63 : 46	45 : 32	1.000

**Table 2:** Haemoglobin levels preoperative and postoperative days 1, 2 and 3 in the heparin, and nonheparinised groups.

Time point	Heparin	No heparin	*P* value
Pre Op	12.4 ± 1.8	12.5 ± 1.3	0.890
Day 1	11.5 ± 1.5*	11.8 ± 1.3*	0.155
Day 2	11.4 ± 1.3	11.7 ± 1.3	0.213
Day 3	11.5 ± 1.3	11.4 ± 1.2	0.424

(*P < 0.05).
